# Influence of Plasmid Type on the Replication of *Rhodococcus equi* in Host Macrophages

**DOI:** 10.1128/mSphere.00186-16

**Published:** 2016-10-12

**Authors:** Jennifer M. Willingham-Lane, Londa J. Berghaus, Steeve Giguère, Mary K. Hondalus

**Affiliations:** a Department of Infectious Disease, University of Georgia, Athens, Georgia, USA; b Department of Large Animal Medicine, University of Georgia, Athens, Georgia, USA; University of Kentucky

**Keywords:** *Rhodococcus equi*, macrophage, species tropism, *vap*

## Abstract

The soil-dwelling, saprophytic actinomycete *Rhodococcus equi* is a multihost, facultative intracellular pathogen of macrophages. When inhaled by susceptible foals, it causes severe bronchopneumonia. It is also a pathogen of pigs, which may develop submaxillary lymphadenitis upon exposure. *R. equi* isolates obtained from foals and pigs possess conjugative plasmids housing a pathogenicity island (PAI) containing a novel family of genes of unknown function called the virulence-associated protein or *vap* family. The PAI regions of the equine and swine plasmids differ in *vap* gene composition, with equine isolates possessing six *vap* genes, including the major virulence determinant *vapA*, while the PAIs of swine isolates house *vapB* and five other unique *vap* genes. Possession of the pVAPA-type virulence plasmid by equine isolates bestows the capacity for intramacrophage replication essential for disease development *in vivo*. Swine isolates of *R. equi* are largely unstudied. Here, we show that *R. equi* isolates from pigs, carrying pVAPB-type plasmids, are able to replicate in a plasmid-dependent manner in macrophages obtained from a variety of species (murine, swine, and equine) and anatomical locations. Similarly, equine isolates carrying pVAPA-type plasmids are capable of replication in swine macrophages. Plasmid swapping between equine and swine strains through conjugation did not alter the intracellular replication capacity of the parental strain, indicating that coevolution of the plasmid and chromosome is not crucial for this attribute. These results demonstrate that while distinct plasmid types exist among *R. equi* isolates obtained from equine and swine sources, this tropism is not determined by host species-specific intramacrophage replication capabilities.

**IMPORTANCE** This work greatly advances our understanding of the opportunistic pathogen *Rhodococcus equi*, a disease agent of animals and immunocompromised people. Clinical isolates from diseased foals carry a conjugative virulence plasmid, pVAPA1037, that expresses Vap proteins, including VapA, essential for intramacrophage replication and virulence *in vivo*. The understudied *R. equi* isolates from pigs carry a related but different plasmid, pVAPB, expressing distinct Vap proteins, including VapB. In this work, we document for the first time that *R. equi* isolates carrying pVAPB-type plasmids are capable of intramacrophage replication. Moreover, we show that *R. equi* isolates carrying either plasmid type can replicate in both equine and swine macrophages, indicating that host species tropism is not due to species-specific intramacrophage replication capabilities defined by plasmid type. Furthermore, plasmid swapping between equine and swine strains did not alter intracellular replication capacity, indicating that coevolution of the plasmid and chromosome is not essential for intracellular growth.

## Abstract (precis)

This work greatly advances our understanding of the opportunistic pathogen *Rhodococcus equi*, a disease agent of animals and immunocompromised people. Clinical isolates from diseased foals carry a conjugative virulence plasmid, pVAPA1037, that expresses Vap proteins, including VapA, essential for intramacrophage replication and virulence *in vivo*. The understudied *R. equi* isolates from pigs carry a related but different plasmid, pVAPB, expressing distinct Vap proteins, including VapB. In this work, we document for the first time that *R. equi* isolates carrying pVAPB-type plasmids are capable of intramacrophage replication. Moreover, we show that *R. equi* isolates carrying either plasmid type can replicate in both equine and swine macrophages, indicating that host species tropism is not due to species-specific intramacrophage replication capabilities defined by plasmid type. Furthermore, plasmid swapping between equine and swine strains did not alter intracellular replication capacity, indicating that coevolution of the plasmid and chromosome is not essential for intracellular growth.

## INTRODUCTION

The genus *Rhodococcus* is a diverse taxon that includes numerous environmental bacteria, many of which are utilized for biotechnological applications (1). There are only two identified pathogenic members of the *Rhodococcus* genus, the plant pathogen *Rhodococcus fascians*, the causative agent of leafy gall disease (2), and the animal and human pathogen *Rhodococcus equi* (3–5). The soil saprophytic bacterium *R. equi* is a facultative intracellular pathogen of macrophages (6, 7). In foals, *R. equi* exposure occurs through inhalation, typically resulting in pneumonia characterized by the formation of pyogranulomatous lesions within the lungs (8, 9). Pigs and cattle are also susceptible to *R. equi* infection. However, in these hosts, the clinical appearance is different from that of foals, with disease typically presenting as submaxillary lymphadenitis and abscessation of the respiratory lymph nodes, respectively (10–12). *R. equi* is also an important opportunistic pathogen of immunocompromised people. In humans, the most common clinical manifestation is necrotizing pneumonia, which is seen in ~80% of immunocompromised patients infected with *R. equi* (13, 14).

All *R. equi* isolates from diseased foals and the majority of those obtained from affected swine and humans carry a large circular, conjugative plasmid (15–19). In contrast, *R. equi* isolates obtained from cattle typically carry a linear plasmid (20). To date, five *R. equi* plasmids have been sequenced and annotated. Two were obtained from strains isolated from pneumonic foals (103S and 33701), one isolate was derived from a person with *R. equi* pneumonia (1593), and the remaining two strains were acquired from heifers with lymphadenitis (PAM1571 and PAM2012) (20–22). Sequence analysis data for the five *R. equi* plasmids showed that the plasmids can be divided into four distinct regions based on gene homology: conjugation, plasmid replication-and-partitioning, unknown function, and a pathogenicity island (PAI) (20–22). Plasmids from the equine isolate 103S and 33701 strains are considered the same element since they are virtually identical in nucleotide sequence and size (80,609 and 80,610 bp, respectively). Both plasmids encode the 17-kDa VapA protein, and these plasmids are referred to as pVAPA1037 (22). The third plasmid, sequenced from the human strain 1593, is 79,251 bp, encodes the 20-kDa antigenic protein VapB, and is known as pVAPB1593 (21). Last, the plasmid sequence of *R. equi* strain 1571, acquired from cattle, is 119,931 bp in length, possesses VapN, and is termed pVAPN1571 (20). *R. equi* virulence plasmids, encoding VapA, VapB, or VapN, are referred to colloquially as being of the pVAPA, pVAPB, or pVAPN type. Interestingly, plasmids derived from equine isolates are of the pVAPA type, plasmids from swine strains are typically of the pVAPB type, and plasmids obtained from bovine isolates are primarily of the pVAPN type. Human *R. equi* isolates carry either the pVAPA- or pVAPB-type plasmid or no plasmid at all (17, 23).

Approximately 75% of the sequence of pVAPA1037 and pVAPB1593 is highly conserved (showing 95% DNA sequence identity). This conserved sequence is referred to as the plasmid backbone and includes the regions of conjugation, of plasmid replication-and-partitioning, and of unknown function (21). The differences found in this backbone sequence are two additional coding sequences (CDSs) encoding putative membrane proteins, which are specific to pVAPB1593, as well as one gene from each plasmid type that is corrupted (21). While the pVAPN1571 linear plasmid also possesses these categorical regions of the plasmid backbone, the genes within these regions are unrelated to those found in pVAPA1037 or pVAPB1593, bearing more similarity to the linear plasmid from the *Rhodococcus* species strain NS1 (20, 24).

In contrast to the high degree of sequence similarity observed in the plasmid backbone of pVAPA1037 and pVAPB1593, the PAI regions share only 43% DNA sequence identity. Despite the divergent sequences, the two PAI regions share certain genetic characteristics, such as the presence of a family of genes known as the virulence-associated protein or *vap* family. The pVAPA1037 plasmid contains 6 full-length *vap* genes (*vapA*, *-C*, *-D*, *-E*, -*G*, and -*H*) along with 3 *vap* pseudogenes (*vapF*, -*I*, and -*X*), pVAPB1593 includes 6 full-length *vap* genes (*vapB*, *-J*, -*K1*, -*K2*, -*L*, and -*M*), and pVAPN1571 contains 4 full-length *vap* genes (*vapN*, *vapO*, *vapP*, and *vapQ*) and 2 pseudogenes (*vapR* and *vapS*) (20–22). Although all *vap* genes share homology to one another, each plasmid type has a distinct *vap* gene composition, which may reflect different niche specificities among the bacterial isolates. Comparative analyses of *vap* gene DNA sequences suggest that the observed differences are a result of gene duplications, translocations, inversions, and insertion/deletion events (21).

All *R. equi* isolates obtained from foals contain the pVAPA-type plasmid, which is required for intracellular replication in *in vitro*-cultured macrophages of equine and murine origin and *in vivo* replication in the equine host and murine infection model (4, 15, 25, 26). The *vap* genes found on this plasmid have been characterized more thoroughly than those of the pVAPB-type plasmid. Of the six full-length *vap* genes found in pVAPA1037, *vapA* has been shown to be a key virulence factor and encodes VapA, the cell envelope-associated protein previously mentioned (27). Through the analysis of a *vapA* deletion mutant strain, Jain and colleagues demonstrated VapA to be essential for the intracellular growth and virulence of *R. equi*. The loss of *vapA* resulted in a fully attenuated mutant, no longer able to replicate in macrophages or establish disease in the *in vivo* chronic disease mouse model (27). The role of VapA in macrophage infection is not fully elucidated, but VapA appears to interfere with normal phagosome maturation (28). *R. equi* isolates containing pVAPB-type plasmids have been demonstrated to cause disease in mice in *in vivo* infection models, although the virulence factors required for disease development have yet to be identified (18). Interestingly, the majority of *R. equi* strains isolated from the lymph nodes of swine typically carry plasmids encoding VapB (10, 29, 30). The observation that all *R. equi* isolates obtained from foals carry a pVAPA-type plasmid and the vast majority of *R. equi* isolates from swine possess a pVAPB-type plasmid led to the question of whether the genetic differences between the equine pVAPA- and swine pVAPB-type plasmids dictate host species tropism, potentially at the level of intramacrophage replication. This work examines whether plasmid type carriage determines an *R. equi* isolate’s ability to replicate within macrophages of distinct host species (murine, equine, and swine).

## RESULTS

### Multiple pVAPB-type plasmid-carrying *R. equi* isolates are capable of replication within murine macrophages.

**FIG 1:**
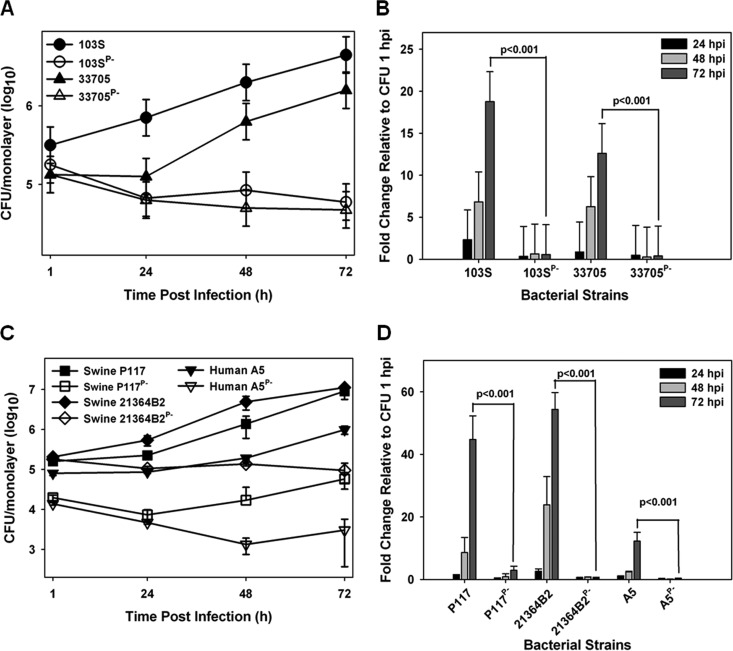
pVAPB-type plasmid-containing *R. equi* isolates can replicate in murine macrophages. The intracellular growth of *R. equi* strains was assessed in murine bone marrow-derived macrophages infected with strains 103S, 103S^P−^, 33705, and 33705^P−^ (A and B) and P117, P117^P−^, 21364B2, 21364B2^P−^, A5, and A5^P−^ (C and D) at an MOI of 10:1. Following a 1-h incubation allowing for phagocytosis, monolayers were washed and medium supplemented with 20 µg/ml amikacin was added to the monolayers to prevent extracellular bacterial growth. Triplicate monolayers were lysed at 24 h, 48 h, and 72 h postinfection (hpi). (A and C) Intracellular growth of *R. equi* strains following infection of macrophages. (B and D) Fold changes in CFU of intracellular bacteria at 24, 48, and 72 hpi relative to 1 hpi. Error bars represent standard deviations from the means. Data with statistical analysis are a compilation of 3 individual experiments.

Currently, there is no published work examining the intramacrophage growth capabilities of *R. equi* strains from swine typically containing the pVAPB-type plasmid. In order to assess whether the observed host species-plasmid type carriage dictates species-specific intramacrophage replication, we first assessed whether a swine *R. equi* isolate (33705) carrying a VapB-type plasmid possessed the ability to replicate within murine macrophages. The murine macrophage model is a well-established *in vitro* model system of intracellular growth that has been shown to correlate with strain virulence (27, 31, 32). *R. equi* isolates carrying the pVAPA-type plasmid are known to be capable of replication within murine *in vitro*-cultured macrophages (4), and therefore, a pVAPA-positive isolate (103S) was utilized as a reference to measure the replicative potential of numerous pVAPB-type plasmid-containing strains. Murine bone marrow-derived macrophages (BMDMs) were infected with plasmid-containing and isogenic plasmid-cured derivatives of the indicated *R. equi* strains, and bacterial intracellular replication was followed by standard lysis and plating of the infected macrophage monolayers over the course of 72 h. As illustrated in Fig. 1A and B, both 103S, a well-characterized equine isolate originally obtained from a foal with *R. equi* pneumonia and carrying pVAPA1037, and the swine isolate 33705, obtained from the lymph node of a pig, replicated in murine macrophages, showing an ~20- and an ~15-fold increase in CFU at 72 h postinfection (hpi) compared to 1 hpi, respectively. In contrast, the isogenic strains lacking a virulence plasmid (strains 103S^P−^ and 33705^P−^) failed to replicate intracellularly. To determine if the replicative ability of 33705 was a strain-specific phenotype, three additional pVAPB-type plasmid-containing isolates were similarly analyzed. The examined isolates were strains P117 and 21364B2, both obtained from pigs, as well as strain A5, a human *R. equi* clinical isolate from a patient with AIDS (18). Notably, strain A5 has been classified as intermediately virulent in mice and foals relative to an equine isolate carrying pVAPA1037. However, its ability to replicate within macrophages had not been examined (18, 33). Plasmid-cured isogenic derivatives of the abovementioned strains, created through repeated subculture, were analyzed in parallel. As observed with the 33705 strain, the other swine isolates and the pVAPB-type plasmid-containing human strain A5 were found capable of intramacrophage replication, and plasmid curing was associated with the loss of replicative ability (Fig. 1C and D). Both swine strains, P117 and 21364B2, replicated ~50-fold over 72 h, whereas the human A5 isolate replicated less efficiently, ~15-fold at 72 hpi. These data show that while there is some degree of strain-to-strain variability in the magnitude of intracellular growth, pVAPB-type plasmid-carrying *R. equi* strains, like equine isolates carrying pVAPA-type plasmids, are able to replicate in murine macrophages and intracellular replication is plasmid dependent.

### The replicative ability of pVAPB-type plasmid-carrying *R. equi* isolates varies in equine macrophages.

**FIG 2:**
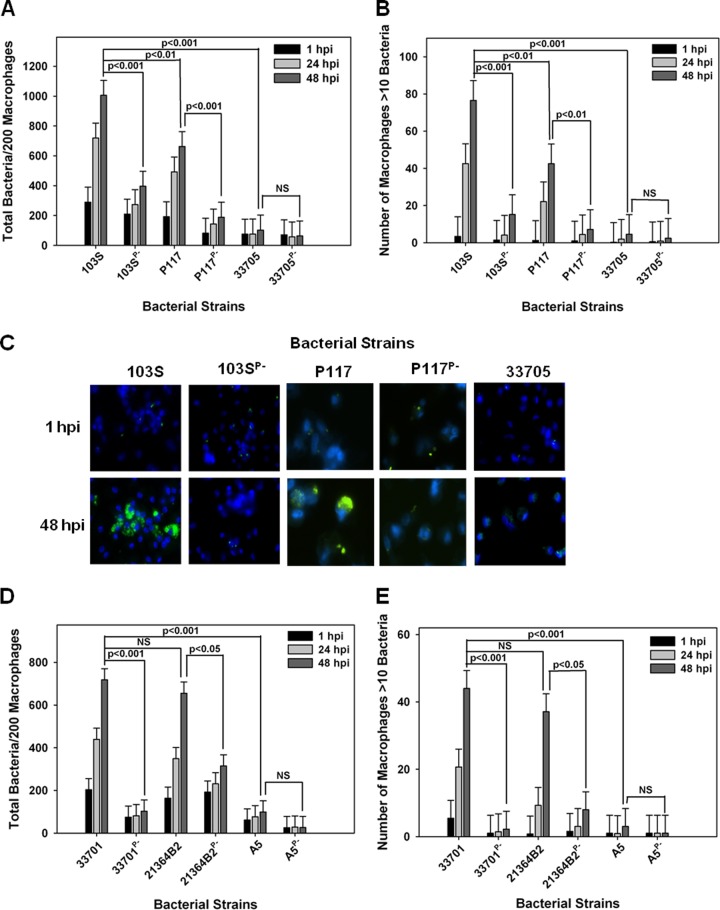
pVAPB-type plasmid-containing *R. equi* isolates display variable replication ability in equine macrophages. The intracellular growth of *R. equi* in equine alveolar macrophages infected with strains 103S, 103S^P−^, P117, P117^P−^, 33705, 33705^P−^, 33701, 33701^P−^, 21364B2, 21364B2^P−^, A5, and A5^P−^ at an MOI of 5:1 was assessed. Following a 1-h incubation allowing for phagocytosis, monolayers were washed and medium supplemented with amikacin was added to the monolayers to kill any extracellular bacteria. Triplicate monolayers were fixed at 1 h, 24 h, and 48 h postinfection (hpi), stained as described in Materials and Methods, and examined under fluorescence microscopy. The number of bacteria per 200 macrophages (A and D) and the number of macrophages with greater than 10 bacteria per 200 macrophages (B and E) were counted. Representative images of the infected macrophage monolayers are shown (×100 magnification) (C). In these representative microscopy images, *R. equi* displays green fluorescence and the macrophage nucleus is DAPI stained. Error bars represent standard deviations from the means. Data and statistical analysis are a compilation of 4 individual experiments. NS, not significant.

Once it was established that *R. equi* isolates possessing pVAPB-type plasmids were capable of replication in murine macrophages, the ability of the swine strains to replicate within equine alveolar macrophages was examined. Macrophages were infected with isolates 33705 and P117, along with their isogenic strains lacking a plasmid. As controls, these macrophages were also infected with the equine pVAPA-containing isolate 103S, known to replicate within these cells, and with its intracellular-growth-impaired plasmid-cured derivative, 103S^P−^ (32). Bacterial replication was examined and quantified by fluorescence microscopy in which the number of bacteria per 200 macrophages was recorded over time in triplicate at each time point. As an additional assessment of intracellular growth, the number of macrophages containing greater than 10 bacteria per 200 macrophages was also recorded as previously described (32). As expected, the equine isolate 103S carrying pVAPA1037 replicated within equine macrophages, whereas its plasmid-cured derivative 103S^P−^ did not (Fig. 2A to C). The swine isolate 33705 failed to grow within equine macrophages regardless of plasmid possession. In contrast, strain P117, also originating from swine, exhibited plasmid-dependent replicative ability in these cells (Fig. 2A to C). Given that the two examined swine isolates demonstrated differences in replicative capacity, the intracellular growth of two additional pVAPB-type plasmid-carrying isolates, one from a pig (strain 21364B2) and the other from a person with *R. equi* infection (A5), was examined. The replication potential of an additional equine isolate, 33701 carrying pVAPA1037, known to cause disease in mice and foals (15, 34), was used as a positive control for intracellular growth. As expected, strain 33701 displayed virulence plasmid-dependent replication within equine macrophages, as did the swine strain 21364B2 (Fig. 2D and E). In contrast, the pVAPB-type plasmid-containing human isolate A5 was unable to replicate within equine macrophages irrespective of plasmid possession, an outcome similar to that displayed by the swine strain 33705. These data presented in Fig. 2 demonstrate that some *R. equi* isolates carrying the pVAPB-type plasmid have the capacity to replicate within equine macrophages but that plasmid possession is not the sole criterion conferring replicative ability, since some pVAPB plasmid-containing isolates, such as 33705 and A5 specifically, lack this capability.

### Examination of the intracellular replicative capacity of pVAPA-type and pVAPB-type plasmid-possessing *R. equi* isolates in swine macrophages.

**FIG 3:**
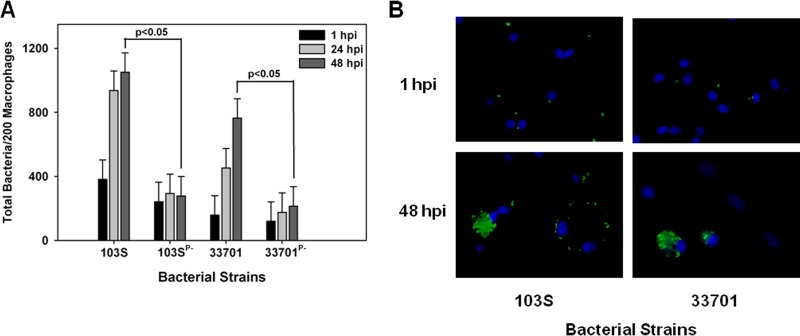
Equine *R. equi* isolates containing the pVAPA1037 plasmids replicate in swine macrophages. The intracellular growth of equine *R. equi* strains 103S, 103S^P−^, 33701, and 33701^P−^ (A and B) was assessed in swine monocyte-derived macrophages infected at an MOI of 5:1. Triplicate monolayers were fixed and stained at 1 h, 24 h, and 48 h postinfection (hpi), and the number of bacteria per 200 macrophages was determined (A). Representative microscopy images of monolayers infected with *R. equi* strains 103S and 33701 at 1 h and 48 h postinfection are shown (×60 magnification) (B). In these images, *R. equi* exhibits green fluorescence and the macrophage nucleus is blue because of DAPI staining. Statistical analysis was performed on the data compiled from 2 individual experiments.

**FIG 4:**
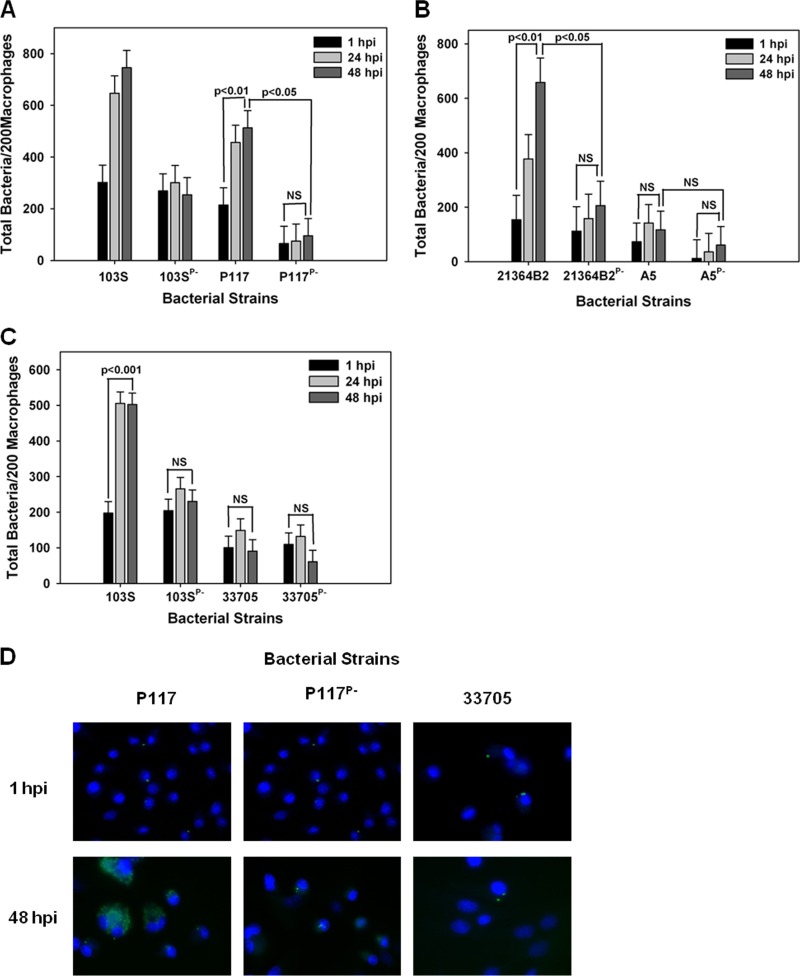
pVAPB-type plasmid-containing *R. equi* isolates display variable replication ability in swine macrophages. Bacterial intracellular growth was assessed in swine monocyte-derived macrophages infected with *R. equi* strains 103S, 103S^P−^, P117, and P117^P−^ (A), 21364B2, 21364B2^P−^, A5, and A5^P−^ (B), and 103S, 103S^P−^, 33705, and 33705^P−^ (C) at an MOI of 5:1. Triplicate monolayers were fixed at 1 h, 24 h, and 48 h postinfection (hpi) and stained. Then, the number of bacteria per 200 macrophages (A, B, and C) was counted using fluorescence microscopy. Representative microscopy images are shown (×60 magnification) (D). Statistical analysis was performed on data compiled from 3 individual experiments. NS, not significant.

We next evaluated the growth potential of the two equine isolates discussed above, 103S and 33701, in swine monocyte-derived macrophages. Interestingly, as shown in Fig. 3A and B, both equine isolates carrying their pVAPA1037 plasmids were able to replicate in swine macrophages in a virulence plasmid-dependent manner. Then, the replicative capacity of the pVAPB-type plasmid-containing *R. equi* isolates was assessed in these cells. Similar to the findings with equine macrophages, we observed variability in the replicative ability among these isolates. For example, swine strains P117 and 21364B2 grew in swine macrophages (Fig. 4A, B, and D). Notably, the intracellular replication displayed by these two strains was plasmid dependent, as plasmid-free derivatives (P117^P−^ and 21364B2^P−^) did not replicate. Much like the results observed with infection of equine alveolar macrophages, the swine strain 33705 (Fig. 4C and D) and the human isolate A5 (Fig. 4B) were unable to grow in swine macrophages despite being able to do so in murine macrophages. Once again, these data showed variable intracellular replicative ability among the pVAPB-type plasmid-containing isolates; however, the strains found capable of intracellular replication in equine macrophages were the same ones demonstrating this capacity in swine macrophages.

### *R. equi* transconjugants demonstrate intracellular growth characteristics consistent with those of the strain’s parental chromosomal background.

**FIG 5:**
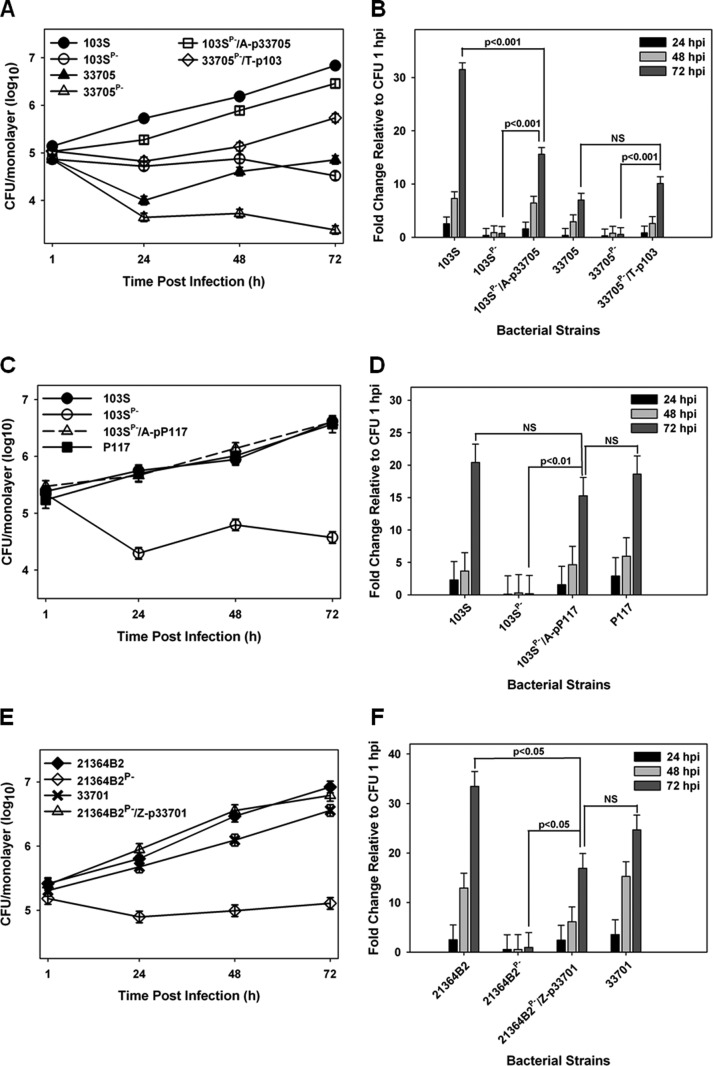
*R. equi* carriage of nonnative plasmids enables replication in murine macrophages. The intracellular growth of *R. equi* transconjugant strains was assessed in bone marrow-derived macrophages infected with 103S, 103S^P−^, 103S^P−^/A-p33705, 33705, 33705^P−^, and 33705^P−^/T-p103 (A and B), 103S, 103S^P−^, 103S^P−^/A-pP117, and P117 (C and D), and 21364B2, 21364B2^P−^, 21364B2^P−^/Z-p33701, and 33701 (E and F) at an MOI of 10:1. Triplicate monolayers were lysed at 24 h, 48 h, and 72 h postinfection (hpi). (A, C, and E) Intracellular growth of *R. equi* strains following infection of macrophages expressed as CFU per monolayer over time. (B, D, and F) Fold changes in CFU of intracellular bacteria at 24, 48, and 72 hpi relative to 1 hpi. Error bars represent standard deviations from the means. Statistical analysis was performed on a compilation of 2 separate experiments. NS, not significant.

As mentioned, it has been reported that *R. equi* plasmid type possession, pVAPA versus pVAPB type, is typically host species specific (10, 11, 15, 16, 26, 29, 34), with pVAPA- and pVAPB-type plasmid carriage characteristic of equine and swine isolates, respectively. A traditional means to determine whether a gene or set of genes is important for host species tropism is to express the gene(s) of interest in a strain of different tropism and evaluate the consequences. Therefore, *R. equi* strains were constructed via conjugation in which pVAPA- and pVAPB-type plasmids were transferred to plasmid-cured derivative recipient strains originating from a different host species (19). For example, 103S^P−^/A, a pVAPA1037-cured derivative of equine origin, was provided the pVAPB-type plasmid from the swine isolate 33705, and a pVAPB-free version of the latter (33705^P−^/T) received the pVAPA1037 plasmid of strain 103S. Complete virulence plasmid transfer was verified through PCR analysis using primer pairs that amplified various regions of the plasmid and also confirmed the presence of either *vapA* or *vapB* (see Fig. S1A to C in the supplemental material) as well as the presence of the chromosomal antibiotic resistance gene used to mark and identify the specific recipient strain (see Fig. S1D). Subsequently, the intracellular growth potential of these transconjugant strains was examined initially in murine macrophages. As demonstrated in Fig. 5A and B, the transconjugant strains 103S^P−^/A-p33705 and 33705^P−^/T-p103 carrying nonnative plasmids were found capable of replication in these cells. However, the transconjugant 103S^P−^/A-p33705 replicated significantly less efficiently than 103S with its native plasmid, with an ~18-fold increase in bacterial numbers compared to an ~30-fold increase in bacterial numbers over 72 hpi, respectively. To further examine the growth potential of *R. equi* isolates in possession of nonnative plasmids, two additional transconjugant strains were created, verified by PCR analysis as previously described, and then similarly examined. The plasmid-cured derivative 103S^P−^/A of equine origin was provided the swine P117 pVAPB-type plasmid (see Fig. S1B), and the plasmid-free product 21364B2^P−^/Z (see Fig. S1C) of swine origin was given the equine 33701 pVAPA1037 virulence plasmid. Similarly, these transconjugants, 103S^P−^/A-pP117 and 2134B2^P−^/Z-p33701, respectively, were found proficient for intracellular replication in murine macrophages (Fig. 5C to F). Cumulatively, these data illustrate that within the murine model system of intramacrophage growth, nonnative-plasmid-type carriage does allow an *R. equi* strain to replicate intracellularly.

10.1128/mSphere.00186-16.1 Figure S1  Analysis of transconjugants created by conjugal transfer of pVAPA-type and pVAPB-type plasmids to plasmid-cured recipient *R. equi* strains of swine and equine origin, respectively. Plasmids were transferred via conjugation by mating unmarked, virulence plasmid-carrying donor strains with chromosomally marked, plasmid-free recipients. Complete plasmid transfer was confirmed in the transconjugants 103S^P−^/A-p33705 and 33705^P−^/T-p103 (A), 103S^P−^/A-pP117 (B), and 21364B2^P−^/Z-p33701 (C) using primer pairs (Table 2) that anneal to various regions along the plasmid backbone in addition to the *vapA* and *vapB* genes. The identity of the recipient background was confirmed via PCR amplification of the correct integrated resistance gene (D). Recipient 103S^P−^/A was marked with an apramycin resistance cassette; recipient 21864B2^P−^/Z was marked with a zeocin resistance gene; recipient 33705^P−^/T was marked via integration of a trimethoprim resistance gene. Standard molecular weight markers (M) are shown in the leftmost lane on all of the gels. Download Figure S1, PDF file, 3.6 MB. Copyright © 2016 Willingham-Lane et al. 2016 Willingham-Lane et al. This content is distributed under the terms of the Creative Commons Attribution 4.0 International license.

**FIG 6:**
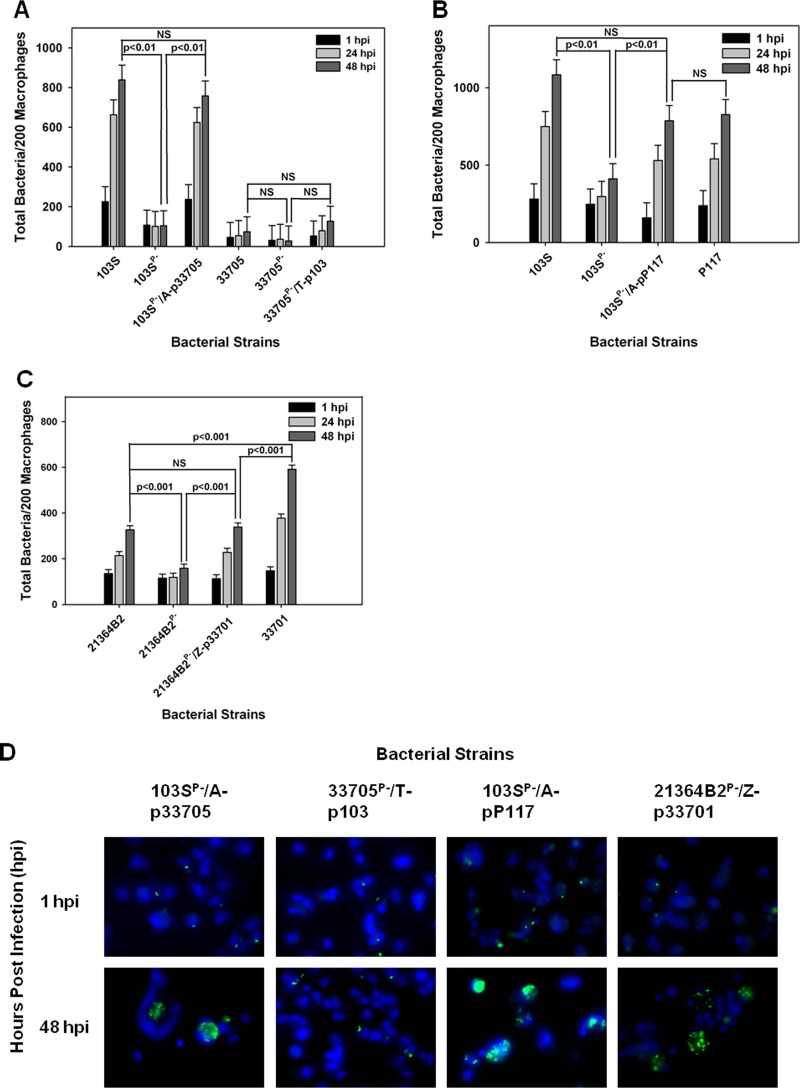
Effect of nonnative plasmid carriage and chromosomal background on *R. equi* intracellular growth phenotype in equine alveolar macrophages. (A to C) The intracellular growth of *R. equi* transconjugant strains was assessed in equine alveolar macrophages infected with 103S, 103S^P−^, 103S^P−^/A-p33705, 33705, 33705^P−^, and 33705^P−^/T-p103 (A), 103S, 103S^P−^, 103S^P−^/A-pP117, and P117 (B), and 21364B2, 21364B2^P−^, 21364B2^P−^/Z-p33701, and 33701 (C) at an MOI of 5:1. Triplicate monolayers were fixed 1 h, 24 h, and 48 h postinfection (hpi) and stained, and the number of bacteria per 200 macrophages was counted under fluorescence microscopy. (D) Representative microscopy images are shown (×60 magnification). Statistical analysis was performed on data compiled from 2 individual experiments. NS, not significant.

We next extended our analysis of the intracellular growth phenotype of the transconjugant strains to equine macrophages. Figure 6A, B, and D illustrates that the equine 103S^P−^ strain possessing a pVAPB-type plasmid from either swine isolate 33705 or P117 replicated in equine macrophages. Likewise, the swine 21364B2^P−^ strain possessing the pVAPA1037 plasmid from the equine strain 33701 (strain 21364B2^P−^/Z-p33701) demonstrated replicative ability in equine macrophages (Fig. 6C and D). In contrast, the swine 33705^P−^ isolate possessing the equine 103S plasmid did not replicate in equine macrophages (Fig. 6A and D). The latter finding is particularly interesting in light of the fact that the wild-type parent 33705 swine isolate carrying its endogenous pVAPB-type plasmid was also unable to multiply within equine cells. These results suggest that some component(s) essential for equine macrophage intracellular replication is lacking in or not induced in the genetic background (i.e., chromosome) of this strain.

**FIG 7:**
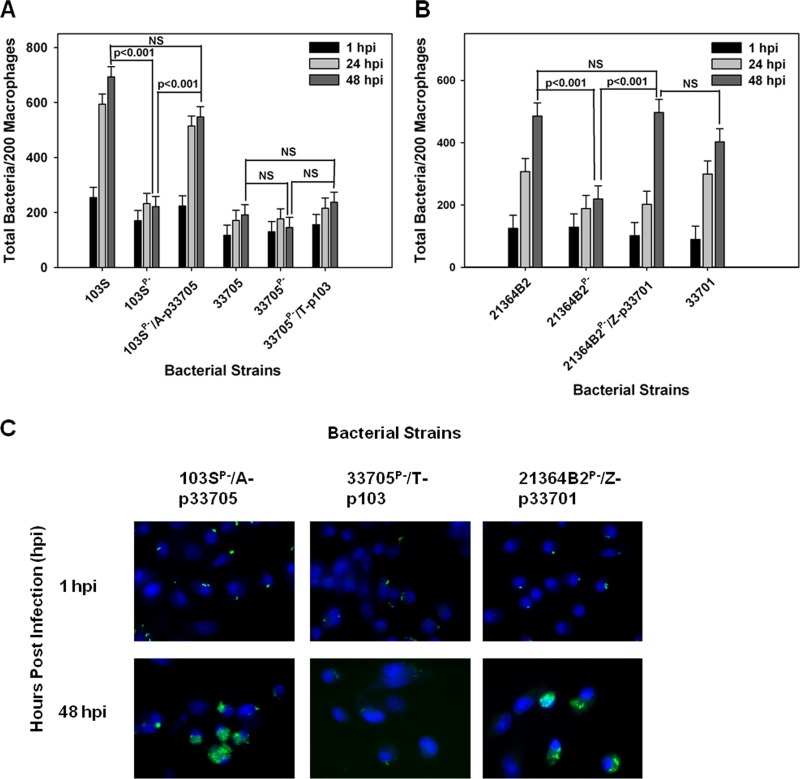
Effect of nonnative plasmid carriage and chromosomal background on *R. equi* growth capabilities in swine macrophages. The intracellular growth of *R. equi* strains carrying native plasmids and transconjugant strains carrying nonnative plasmids was assessed in swine monocyte-derived macrophages infected with 103S, 103S^P−^, 103S^P−^/A-p33705, 33705, 33705^P−^, and 33705^P−^/T-p103 (A) and 21364B2, 21364B2^P−^, 21364B2^P−^/Z-p33701, and 33701 (B) at an MOI of 5:1. Following a 1-h incubation allowing for phagocytosis, monolayers were washed and medium supplemented with amikacin was added to kill any extracellular bacteria. Triplicate monolayers were fixed at 1 h, 24 h, and 48 h postinfection (hpi) and stained, and the number of bacteria per 200 macrophages (A and B) was determined. Representative microscopy images are shown (×60 magnification) (C). Statistical analysis was performed on data compiled from 2 individual experiments. NS, not significant.

Last, we assessed the intracellular growth capabilities of the transconjugants in swine macrophages. As shown in Fig. 7A and C, the plasmid-cured strain of equine origin, 103S^P−^/A, in possession of a pVAPB-type plasmid transferred from the swine isolate 33705 replicated in these macrophages as efficiently as the parent strain (103S) carrying its native pVAPA1037 plasmid. Similarly, the swine 21364B2^P−^/Z isolate carrying the equine 33701 pVAPA1037 plasmid displayed intracellular growth equivalent to that of swine isolate 21364B2 containing its native plasmid (Fig. 7B and C). Like the findings observed in equine macrophages, transconjugant strain 33705^P−^/T-p103, originally of swine origin carrying the 103S pVAPA1037 equine plasmid, was unable to grow within swine macrophages (Fig. 7A and C). In sum, the findings that the transconjugant strains 103S^P−^/A-p33705 and 21364B2^P−^/Z-p103 can replicate in swine macrophages confirm once again that nonnative plasmid carriage can promote the intracellular growth of some *R. equi* strains and that coevolution of the plasmid and chromosome is not essential for this trait. The observation that strain 33705^P−^/T-p103 cannot replicate in these cells implies that the chromosomal background also plays a critical role in determining a given *R. equi* isolate’s ability to survive and grow in macrophages. Cumulatively, these data show that the host species tropism displayed by pVAPA- and pVAPB-type plasmid-containing *R. equi* isolates cannot be explained by species-specific intramacrophage replication.

## DISCUSSION

It has long been acknowledged that clinical isolates of the facultative intracellular bacterium *R. equi* obtained from foals with pneumonia and associated disease manifestations carry an ~80-kb virulence plasmid (pVAPA1037) and express the crucial virulence determinant VapA (15, 16, 34). Also well established is the finding that the majority of *R. equi* isolates from swine carry a highly related but different plasmid (pVAPB-type) and express Vap proteins distinct from VapA, including VapB (10, 11, 21, 29). We hypothesized that particular plasmid types were associated with specific hosts because plasmid type allowed for intramacrophage replication capabilities in a host-specific manner. Interestingly, the data presented here reveal that plasmid type does not confer species-specific macrophage replication abilities. *R. equi* isolates possessing either pVAPA-type or pVAPB-type plasmids are capable of replication within macrophages derived from various host species, including the mouse, horse, and pig.

Since it appears that *R. equi* has the capacity to replicate in macrophages regardless of plasmid type or macrophage species, it raises the question why pVAPB-type plasmid-carrying *R. equi* strains are not isolated from diseased foals. A simple explanation is that foals are not exposed to strains of *R. equi* that carry this plasmid type. Currently, there is a paucity of knowledge regarding the epidemiology of *R. equi* isolates carrying pVAPB-type plasmids. Despite the large number of studies examining the presence of *R. equi* in the soil and its impact on foal disease, the majority of studies have simply determined plasmid possession through the presence or absence of *vapA* (35–40). Other plasmid components, including the conserved gene *traA*, are rarely screened for, but if that were done, it could identify *R. equi* isolates carrying various plasmid types, and additional screening could be used to determine specific plasmid type (41). Occasionally, studies have analyzed soil samples for the presence of both pVAPA- and pVAPB-type plasmids, and interestingly, most of the collected *R. equi* isolates are negative for both types (42, 43). In Hungary, Major and colleagues analyzed 48 soil samples from stud farms for *vapA* and *vapB*, where 54.2% of the isolates were *vapA* positive and the remaining were negative for both genes (44). If these results are generally representative of the *R. equi* populations found in the soil of horse farms worldwide, the absence of foals infected with *R. equi* strains possessing pVAPB-type plasmids could be due to a lack of exposure of the foal to bacteria carrying this plasmid type and not because of an inability of such a bacterium to cause disease in the equine host. pVAPB-type plasmid-carrying *R. equi* strains have been isolated from the soil of pig farms (23); however, very little is known about the frequency at which *R. equi* isolates containing pVAPB-type plasmids are found in the environment in general or their environmental stability, especially within the environment of foals.

Given that *R. equi* strains carrying pVAPB-type plasmids have the ability to replicate in equine alveolar macrophages, it would be of interest to perform a head-to-head challenge experiment in foals wherein the disease-causing capacity of a pVAPA-type-possessing *R. equi* strain is directly compared to that of an *R. equi* strain carrying a pVAPB-type plasmid. Such a study was attempted several years ago (34). In this study, two foals were intratracheally infected with either 10^5^ CFU or 10^6^ CFU of the clinical equine isolate 33701, containing pVAPA1037 (33). Two other foals were similarly infected with *R. equi* strain A5, the human isolate possessing pVAPB, at a challenge dose of 10^6^ or 10^9^ CFU, respectively. Both foals infected with 33701, at either bacterial dose, developed severe clinical signs, including depression, anorexia, and pyrexia (40.5°C), at days 12 to 14 postchallenge. The foal challenged with 10^6^ CFU of *R. equi* strain 33701 succumbed to infection on day 22 postinfection. In contrast, the foal infected with 10^6^ CFU of *R. equi* pVAPB-type plasmid-possessing strain A5 displayed no clinical signs at any time, and *R. equi* was not isolated from tracheal washes or fecal samples. The foal infected with a 1,000-fold-higher bacterial dose of strain A5, 10^9^ CFU, did develop clinical signs, including pyrexia (40°C) and depression, at day 21 postinfection, but symptoms were far less severe than those of the foals infected with a much lower dose of the pVAPA1037-containing *R. equi* isolate. These data implied that an *R. equi* isolate in possession of a pVAPB-type plasmid is less virulent in foals than strains of *R. equi* carrying a pVAPA-type plasmid. Interestingly, the results of our experiments presented here demonstrate that the pVAPB-containing isolate A5 is less efficient at replicating within murine macrophages than other plasmid-containing *R. equi* isolates in possession of either a pVAPA- or pVAPB-type plasmid. Moreover, we found it completely unable to replicate within equine alveolar or monocyte-derived swine macrophages. Our data provide an explanation for why the A5 strain was unable to establish an infection in foals unless a high inoculum was used, essentially overpowering the biological or immunological system. We speculate that if another pVAPB-type plasmid-containing *R. equi* strain (for example, strain 21364B2 or P117) that displayed replicative ability in equine alveolar macrophages were utilized in a similar challenge of foals, then overt disease might develop at an infection dose similar to that observed in foals challenged with a clinical *R. equi* isolate possessing the pVAPA-type plasmid.

In the event that *R. equi* isolates carrying a pVAPB-type plasmid are found in the environment, foals exposed to these isolates may develop characteristic *R. equi* pneumonia but are treated without confirmative plasmid type carriage determination by diagnostic microbiology laboratories. Additionally, microbiological identification of *R. equi* can be and often is done without assessment of *vap* gene possession; therefore, it is possible that microbiology laboratories are not recognizing pVAPB-type plasmid possession by *R. equi* isolates in clinical samples from foals. Alternatively, *R. equi* carriage of a pVAPB-type plasmid may lead to self-resolving subclinical disease in foals. Ultrasonography has been a method utilized to identify *R. equi* disease prior to clinical presentation (45–47). During these studies, it was determined that the majority of foals that developed ultrasonographic evidence of pulmonary *R. equi* disease resolved the infection prior to the development of clinical disease presentation (47). It is feasible that *R. equi* isolates in possession of either a pVAPA- or a pVAPB-type plasmid are capable of establishing an infection but that only those strains in possession of a pVAPA-type plasmid progress to clinical disease in foals.

Another potential explanation of why *R. equi* strains carrying pVAPB-type plasmids are not isolated from foals is that these strains do not establish infection and produce disease in foals in the same manner as do pVAPA-containing strains. Perhaps, *R. equi* possessing a pVAPB-type plasmid causes a disease state in foals with a presentation more like that observed in swine. Swine are frequently clinically asymptomatic with lymph node abscessation revealed only upon postmortem examination at slaughter. Since the submandibular lymph nodes of seemingly healthy foals are not routinely examined for the presence of *R. equi*, if disease were located there it could be missed altogether. Additionally, as the foal ages and becomes less immunologically naive, presumably the pVAPB-containing isolate, if present, would be cleared.

Little work has been done addressing the pathogenesis of *R. equi* disease in pigs. However, one study examined the pathogenicity of the pVAPA1037-containing equine clinical *R. equi* isolate 33701 and the human clinical *R. equi* isolate, A5, in possession of a pVAPB-type plasmid in pigs. Pigs were challenged in one of two ways. The first was a single intravenous (ear vein) dose of 10^8^ or 10^9^ CFU of *R. equi* strain 33701 or A5 in possession of the pVAPA1037- or pVAPB-type plasmid, respectively, along with the plasmid-cured derivative of 33701 given at an inoculum of 10^9^ CFU (48). In the second challenge experiment, pigs were administered two bacterial doses of 10^9^ CFU of *R. equi* strain 33701, A5, or the plasmid-cured 33701 by intramuscular injection, given 7 days apart (48). Overall, regardless of plasmid type possession or route of infection, none of the pigs developed clinical signs beyond a transient low-grade fever and weight loss. Interestingly, upon examination of the group of intramuscularly challenged pigs, bacterial recovery from the mandibular lymph nodes of the pig inoculated with the pVAPB plasmid-containing *R. equi* isolate was higher than that from the pig inoculated with the *R. equi* strain carrying the pVAPA1037-type plasmid, specifically 6 × 10^6^ compared to 1 × 10^5^ CFU/g of tissue, respectively (48). Additionally, the plasmid-cured derivative of the equine strain 33701 was not isolated from the mandibular lymph nodes of pigs challenged with this *R. equi* strain (48). All other organs obtained from these pigs were negative for all three bacterial strains. This may suggest that there is a preference for the establishment of an infection by *R. equi* isolates carrying a pVAPB-type plasmid in the mandibular lymph nodes of swine. It would be intriguing to know if a pVAPB-containing *R. equi* isolate shown to replicate in swine macrophages (*R. equi* strain 21364B2 or P117) would produce more dramatic results.

It has been demonstrated that VirR, VirS, and VapA are the only plasmid-encoded proteins on the pVAPA1037-type plasmid required for intracellular replication in murine macrophages (49). The presence of VapA, the 17-kDa protein product of the *vapA* gene, is thought to interfere with normal phagosomal acidification, thereby creating a vacuolar environment conducive to bacterial intracellular survival and replication (4, 28, 50, 51). Importantly, VirR and VirS not only regulate *vapA* expression but also influence the expression of ~18% of chromosomal genes, having a large impact on the regulatory network of the cell and modulating bacterial physiology to allow adaption to the macrophage environment (49). It has been suggested that acquisition of the horizontally acquired PAI region was advantageous for *R. equi* to survive interactions with predatory protozoa found in abundance in the soil and water (49, 52). While initially VirR and VirS likely minimally influenced the bacterial chromosome, over time and through evolutionary pressure the chromosomal binding efficiency of these proteins improved and their binding was a pivotal event promoting intracellular survival and replication. It is of relevance that the pVAPB-type plasmid carries homologs of the pVAPA-type VirR and VirS proteins, specifically pVAPB1593_0480 and pVAPB1593_0530, which share 92% and 86% amino acid sequence identity, respectively (21). Due to the high degree of sequence similarity, it is likely that the pVAPB-type plasmid-encoded VirR and VirS homologs function similarly and engage in cross talk with the bacterial chromosome to enable intramacrophage survival. However, the differences in protein sequence of these homologs could impart alterations in chromosomal gene regulation between an *R. equi* isolate possessing the pVAPA-type plasmid and one possessing the pVAPB-type plasmid, and such differences in cross talk could contribute to the host species-specific niche of the equine and swine isolates *in vivo*.

Despite the coevolution of the PAI-encoded regulators with a given bacterial chromosome, virulence plasmid swapping via conjugation showed that intramacrophage growth was possible in a strain carrying a nonnative plasmid of a different host species origin. In addition, it was shown that the replicative ability of an isolate is affected by the chromosomal background, wherein genes essential for this trait are known to reside (53–56). Chromosomal influence was illustrated through the examination of several transconjugant strains, for example, strain 103S^P−^/A-p33705, the equine 103S^P−^ strain housing a pVAPB-type plasmid transferred from the swine isolate 33705. 103S^P−^ with its native pVAPA1037-type plasmid, known as 103S, was able to replicate within all three macrophage species analyzed. In contrast, the swine isolate 33705 demonstrated growth only in murine macrophages, lacking the capacity to replicate in macrophages of both equine and swine origin. Intriguingly, the transconjugant 103S^P−^/A-p33705 displayed an intracellular growth phenotype like that of 103S carrying its native VAPA1037-type plasmid, suggesting that the swine pVAPB-type plasmid from 33705 is fully functional, and the inability of this swine isolate to replicate in equine and swine macrophages aligns with the chromosomal background. This observation was further exemplified by analysis of the reverse transconjugant, 33705^P−^/T-p103, a strain that, like 33705, was unable to replicate in equine and swine macrophages, once again showing the influence of the bacterial chromosome.

To date, only one *R. equi* chromosome sequence, that of strain 103S of equine origin, has been published. The availability of additional chromosomal sequences of both equine and swine isolates would be informative and might reveal features specific to *R. equi* isolates possessing different plasmid types. While these chromosomal characteristics may not directly influence bacterial replication within the macrophage, they may nonetheless play a role *in vivo* in the host. For example, differences in gene expression among *R. equi* isolates could result in varied *in vivo* host cytokine induction profiles that affect microbial survival, thus contributing to the observed host species tropism.

In conclusion, *R. equi* strains possessing either a pVAPA- or pVAPB-type plasmid are capable of replication in the three examined macrophage species (murine, equine, and swine). This work suggests that the apparent host species tropism of *R. equi* is not expressed at the level of the intramacrophage replication and is determined by an as-yet-to-be-defined bacterium-host interaction not observable through the analyses performed here. Additionally, through the examination of various transconjugant strains, it was shown that the chromosomal background influences the degree of replicative ability of the bacterial strain regardless of plasmid type, findings which further support previous work showing the profound impact of cross talk between the virulence plasmid and chromosome (49).

## MATERIALS AND METHODS

### Bacterial strains and growth conditions.

*R. equi* strains used in this study are listed in Table 1. *R. equi* 103S was originally isolated from a foal with *R. equi* pneumonia. ATCC 33701 and 33705 were obtained from the American Type Culture Collection (ATCC, Manassas, VA) and were originally isolated from a foal and pig, respectively, with *R. equi* disease. Strains P117 and 21364B2 were isolated from individual pigs, whereas A5 was obtained from a person with *R. equi* pneumonia, and all were a gift from John Prescott (University of Guelph). Plasmid-cured derivatives of the abovementioned *R. equi* strains, 103^P−^ (57), 33701^P−^, 33705^P−^, P117^P−^, 21364B2^P−^, and A5^P−^, were created by serial subculture at 37°C until the virulence plasmid was lost. Virulence plasmid loss was confirmed by PCR analysis using primer pairs (Table 2) which anneal to various regions of the plasmid. Bacterial strains were grown at either 30°C or 37°C (shaking, 200 rpm) in brain heart infusion (BHI) broth. When necessary, antibiotics were added at the indicated concentrations: apramycin (Apr), 80 µg/ml; trimethoprim (Trim), 50 µg/ml; zeocin (Zeo), 50 µg/ml.

**TABLE 1:** Bacterial strains and plasmids

Strain or plasmid	Genotype or characteristic	Reference or source
*Rhodococcus equi* strains		
103S	Wild-type strain with virulence plasmid pVAP1037, expressing *vapA*; originally isolated from a pneumonic foal	31
103S^P−^	Plasmid-cured variant of 103S	57
103S^P−^/A	103^P−^ containing an *aac(3)-IV* gene integrated on the chromosome; Apr^r^	19
33701	Wild-type strain with virulence plasmid pVAP1037, expressing *vapA*; originally isolated from a pneumonic foal	ATCC
33701^P−^	Plasmid-cured variant of 33701	19
33705	*R. equi* strain with pVAPB-type virulence plasmid originally isolated from the lymph node of a pig	ATCC
33705^P−^	Plasmid-cured variant of 33705	19
33705^P−^/T	33705^P−^ containing a *dhfr* gene of mouse origin integrated on the chromosome; Trim^r^	This study
P117	*R. equi* strain with pVAPB-type virulence plasmid originally isolated from the lymph node of a pig	Gift from John Prescott, University of Guelph
P117^P−^	Plasmid-cured variant of P117	This study
21364B2	*R. equi* strain with VAPB-type virulence plasmid originally isolated from a pig	Gift from John Prescott, University of Guelph
21364B2^P−^	Plasmid-cured variant of 21364B2	This study
21364B2^P−^/Z	21364B2^P−^ containing the *ble* gene of *Streptoalloteichus hindustanus* integrated on the chromosome; Zeo^r^	This study
A5	*R. equi* strain with virulence plasmid pVAPB1593 originally isolated from a person with *R. equi* pneumonia	33
A5^P−^	Plasmid-cured variant of A5	This study
103S^P−^/A-p33705 TC	Transconjugant of strain 103^P−^/A and strain 33705, carrying the pVAPB-type plasmid from 33705; Apr^r^	This study
103S^P−^/A-pP117 TC	Transconjugant of strain 103^P−^/A and strain P117, carrying the pVAPB-type plasmid from P117; Apr^r^	This study
33705^P−^/T-p103 TC	Transconjugant of strain 33705^P−^/T and 103S, carrying the pVAPA1037 plasmid from 103S; Trim^r^	This study
21364B2^P−^/Z-p33701 TC	Transconjugant of strain 21364B2^P−^/Z and 33701, carrying the pVAPA1037 plasmid from 33701; Zeo^r^	This study
		
Plasmids		
pSET152	*aac(3)-IV* phiC31 integrase *attP*; Apr^r^	58
pVM6	pSET152 with *dhfr* gene of mouse origin; *aac(3)-IV* removed; phiC31 integrase *attP*; Trim^r^	19
pSET152.zeo.1	pSET152 with *ble* gene of *Streptoalloteichus hindustanus*; Apr^r^ Zeo^r^	This study
pSET152.zeo	pSET152.zeo.1 lacking *aac(3)-IV*; Zeo^r^	This study

**TABLE 2:** Primers utilized for *R. equi* strain confirmation

Name	Sequence (5′ to 3′)	Reference
REVP1	GGAAGGAATGGCAAGAAA	27
REVP1c	TGTGCCGCTTCAAAGGCT	27
REVP6	GAGAGTTCAGTTTCGCGG	27
REVP6c	CCTTTCCATTGGTGTCTTC	27
REtrbA1	GCGTCAGTGCGACAGTGATG	27
REtrbA1c	TCGGAGTCAGGTCGGAGG	27
vapB-F	GGCAGTGCCCTTCTTAAGGATG	This study
vapB-R	AACTGCAGGGGCCTGGATATGG	This study
vapA-F	GACCATGGAGACCGTTCTTGATTCCGGTAG	This study
vapA-R	CTAGGCGTTGTGCCAGCTAC	This study
Zeocin-F	AGTTGACCAGTGCCGTTCC	This study
Zeocin-R	CACGAAGTGCACGCAGTTG	This study
Apramycin-F	GGCCACTTGGACTGATCGAG	This study
Apramycin-R	GCATGACCGACTGGACCTTC	This study
Trimethoprim-F	GCCGGGATCCAGTAAAGTAGACATGGTTTG	19
Trimethoprim-R	CGCTGCAGGTCTTTCTTCTCGTAGACTTC	19

### Plasmid construction.

To mark the chromosome of recipient cells, a new version of the previously described integrating vector pSET152 (58) was created, wherein the apramycin resistance gene was replaced by the *ble* gene of *Streptoalloteichus hindustanus*, providing resistance to zeocin. To do this, pSET152 was digested with XbaI and EcoRV, resulting in a linearized 5,689-bp DNA fragment. The pEM7/Zeo plasmid (Thermo Scientific) was similarly digested, leading to the isolation of the zeocin resistance cassette under the EM7 bacterial promoter (477 bp). The zeocin resistance cassette was ligated with the linearized pSET152 vector, creating pSET152.zeo.1. For removal of the apramycin resistance cassette, pSET152.zeo.1 was digested with SacI, producing a 5.4-kb and a 751-bp DNA fragment. The larger fragment was then self-ligated, generating pSET152.zeo.

### Creation of transconjugants through bacterial conjugation.

Mating was performed as described by Tripathi et al. (19); several *R. equi* strains were marked via the insertion of an integrating vector carrying a specific and varied antibiotic resistance cassette (Table 1). The virulence plasmid-cured 103^P−^ derivative was chromosomally marked with the gene *aac(3)-IV* providing apramycin resistance (designated Apr^r^ or A). Similarly, the *R. equi* strain 33705^P−^ was marked via insertion of a dihydrofolate reductase (*dhfr*) gene conferring trimethoprim resistance (designated Trim^r^ or T) (19). Strain 21364B2^P−^ was marked with the *ble* gene of *Streptoalloteichus hindustanus*, providing resistance to zeocin (designated Zeo^r^ or Z). To facilitate virulence plasmid transfer, equal numbers of chromosomally marked recipient (103^P−^/A, 33705^P−^/T, or 21364B2^P−^/Z) and unmarked donor (103S, 33705, P117, or 33701) strains were used. The donor and recipient strains were grown overnight at 37°C in BHI broth with the recipient cultures supplemented with the appropriate antibiotic; the next day, the optical density at 600 nm (OD_600_) was adjusted to 1.0 (~2 × 10^8^ CFU/ml). Approximately 10^7^ CFU of both donor and recipient bacteria was centrifuged together, resuspended in a small volume (5 to 10 µl), and spotted on BHI agar, and the plates were incubated for 72 h at 30°C. Afterward, the cell mixture was scraped from the plates and resuspended in 1 ml phosphate-buffered saline (PBS). Serial dilutions (up to 1:10^−7^) of the resuspended cells were plated on agar containing the appropriate antibiotics for selection of putative transconjugants as well as recipients. A mixture of colonies from these plates was used to infect murine macrophages as described below in order to amplify and select the lesser-represented transconjugant over the plasmid-cured recipient. At 48 h postinfection, the macrophage monolayer was lysed, the lysate was plated on BHI agar with the appropriate antibiotic, and the plates were incubated for 48 h. Resultant putative transconjugant colonies were screened for the existence of the transferred virulence plasmid using PCR analysis, confirming the presence of the replication/partitioning (primers REVP1/c [Table 2]), conjugation (primers REVP6/c [Table 2]), unknown function (primers REtrbA1/c [Table 2]), and pathogenicity (primers vapA-F and vapA-R or vapB-F and vapB-R [Table 2]) regions as well as the appropriate antibiotic resistance marker (Table 2).

### Bone marrow-derived macrophages.

To obtain macrophage precursors, the marrow of femurs and tibias of female BALB/c mice was flushed into a 50-ml conical tube using cold cation-free phosphate-buffered saline (PBS) supplemented with penicillin G (100 U/ml)-streptomycin (100 µg/ml) (PGS). The cells were centrifuged at 260 × *g* for 10 min at 4°C. The cell pellet was then resuspended in complete medium consisting of Dulbecco modified Eagle medium (DMEM) containing 10% fetal bovine serum (FBS), 10% colony-stimulating factor 1 (CSF-1) from CSF-1-producing L929 cells, and 2 mM glutamine. Cells were washed by centrifugation at 260 × *g* for 10 min. The final resuspension of cells was done in complete medium with 24 ml per mouse used. Then, the precursor cells were plated in 6-well non-tissue-culture-treated plates (4 ml per well) and incubated at 37°C with 5% CO_2_. On the third day, 4 ml of complete medium was added to each well. On day 5, the medium was removed and replaced with complete medium without antibiotic; then, on day 6, the medium was aspirated and each well was washed with 4 ml PBS to remove any nonadherent/dead cells. Then, 4 ml of cold, cation-free PBS was added and the plates were refrigerated at 4°C for 15 min. Afterward, the cells were collected, quantified, and seeded into tissue-culture-treated 24-well plates at a concentration of 2 × 10^5^ per well.

### Equine alveolar macrophages.

To acquire alveolar macrophages, a bronchoalveolar lavage (BAL) was performed on adult horses sedated with xylazine hydrochloride (0.5 mg/kg of body weight intravenously [i.v.]) and butorphanol tartrate (0.02 mg/kg i.v.). A sterile BAL catheter was passed via the nasal cavity and wedged within a bronchus. Four aliquots of 60 ml (~240-ml total) of sterile physiologic saline (0.9% NaCl) solution was infused into the horse’s lungs and aspirated immediately. Once collected, the BAL fluid was centrifuged at 260 × *g* for 10 min at 4°C, and the pellet was resuspended in 50 ml PBS followed by another similar centrifugation. This washing step was repeated two additional times. The final cell pellet was resuspended in minimum essential medium alpha (MEMα) supplemented with 10% donor horse serum (DHS) (Thermo Scientific), 2 mM glutamine, and PGS. Following quantification, 4 × 10^5^ cells were pipetted into each well of a 24-well tissue culture plate, wherein each well contained a 13-mm glass coverslip. The plates were incubated at 37°C with 5% CO_2_ for 4 h to allow for macrophage adherence. After 4 h, the wells were washed 3 times with MEMα to remove nonadherent cells and then the medium was replaced with antibiotic-free MEMα plus 10% DHS plus 2 mM glutamine and the cells were incubated overnight at 37°C with 5% CO_2_.

### Porcine monocyte-derived macrophages.

From each pig, 50 ml of blood was drawn and placed into a collection vial containing 158 USP units of sodium heparin. The blood was diluted with an equal volume of PBS and layered over Ficoll (15 ml Ficoll per 35 ml of blood-PBS) and then centrifuged at 700 × *g* for 30 min at 4°C without braking to allow for proper Ficoll gradient formation. After centrifugation, the buffy coat layer, containing leukocytes, was transferred to a new sterile 50-ml conical tube which was then filled to a final volume of 35 ml with PBS and centrifuged at 260 × *g* for 15 min at 4°C. Following centrifugation, the supernatant was discarded and the cells were resuspended in 35 ml PBS supplemented with PGS and centrifuged two additional times. After the final wash, the cells were quantified and resuspended at a final concentration of 5 × 10^6^ per ml in DMEM supplemented with 10% FBS, 10% CSF-1, 2 mM glutamine, and PGS. The cell suspension was transferred to a 75-cm^2^ tissue-culture-treated flask (5 × 10^7^ cells per flask) and incubated at 37°C with 5% CO_2_ overnight. The next day, the medium was gently removed and replaced with fresh medium supplemented with PGS. The cells were incubated in antibiotic-containing medium for 3 days, and then the medium was replaced with non-antibiotic-containing DMEM supplemented with 10% FBS and 2 mM glutamine. The non-antibiotic-containing medium was removed and replaced with fresh medium at least once more prior to macrophage harvesting on day 7 of culture. For recovery, cells were washed once with PBS and incubated at 37°C for 5 min in PBS supplemented with 1 mM EDTA. Following collection and centrifugation at 4°C, the cells were resuspended in DMEM supplemented with 10% FBS and 2 mM glutamine. Cells were placed in a 24-well tissue culture plate containing 13-mm glass coverslips at a concentration of 2 × 10^5^ cells per well.

### Bacterial intracellular growth assay.

Overnight bacterial broth cultures were grown to an optical density at 600 nm (OD_600_) of 1.0 (~2.0 × 10^8^ CFU/ml), washed once with PBS, and resuspended to the original culture volume in PBS. Macrophage monolayers (bone marrow-derived macrophages [BMDMs] and equine and porcine macrophages) were washed once with warm DMEM. The medium was replaced with DMEM supplemented with 10% FBS and 2 mM glutamine (equine macrophages) and 10% FBS, 10% CSF-1, and 2 mM glutamine (BMDMs and porcine macrophages). Bacteria were added at a multiplicity of infection (MOI) of 5 to 10 bacteria per macrophage. Following 60 min of incubation at 37°C to allow for bacterial binding and uptake, the monolayers were washed 3 times with DMEM to remove any unbound bacteria and the appropriate medium containing 20 µg/ml of amikacin sulfate was added to the monolayers in order to prevent extracellular bacterial replication. At various times postinfection, the macrophage monolayers were washed repeatedly and lysed by the addition of 500 µl of sterile water, and the lysate was collected and plated onto BHI agar. The number of CFU associated with the macrophage lysate was determined after a 48-h incubation at 37°C (BMDMs). Alternatively, the monolayers were fixed with cold 100% methanol for 30 min at 4°C and the associated bacteria were stained with polyclonal rabbit anti-*R. equi* antibody followed by a fluorescein isothiocyanate (FITC)-labeled goat anti-rabbit secondary antibody allowing for the enumeration of the bacteria under fluorescence microscopy.

### Fluorescent staining of *R. equi*-infected macrophages.

Macrophage monolayers on glass coverslips were fixed with 100% methanol for 30 min at 4°C and then washed once with PBS. Then, primary polyclonal rabbit anti-*R. equi* antibody, diluted 1:1,000 in PBS containing 5% normal goat serum (NGS), was added to the fixed monolayers and incubated for 60 min at room temperature (RT). Following washing with PBS containing 5% NGS, goat anti-rabbit antibody conjugated with Alexa Fluor 488 (diluted 1:1,000 in PBS with 5% NGS) was added and the monolayers were incubated for 60 min at RT. Following washing with PBS containing 5% NGS, the coverslips were mounted onto microscope slides using ProLong Gold containing 4′,6-diamidino-2-phenylindole (DAPI) stain (Invitrogen).

### Statistical analysis.

Normality of the data and equality of variances were assessed using the Shapiro-Wilks and Levene tests, respectively. The effects of bacterial strain, time, and interactions between bacterial strain and time on intracellular *R. equi* were assessed using two-way repeated-measures analysis of variance (ANOVA) or with mixed-effects linear modeling with experiment modeled as a random effect and bacterial strain, time, and two-way interactions modeled as fixed nominal factors. When indicated, multiple pairwise comparisons were done using the Holm-Sidak method. Significance was set at a *P* value of <0.05. Statistical analyses were performed using the SigmaPlot (Systat Software, San Jose, CA) or SPSS (IBM SPSS Statistics for Windows, Version 23.0; IBM Corp., Armonk, NY) statistical package.

### Accession number(s).

The pSET152.zeo cloning vector sequence has been deposited into GenBank under accession number KX709879.
