# Supplementary figures and images for: Influence of Plasmid Type on the Replication of Rhodococcus equi in Host Macrophages
Source: mSphere. 2016 Oct 12;1(5):e00186-16. doi: 10.1128/mSphere.00186-16 (PMC5061997; doi:10.1128/mSphere.00186-16)

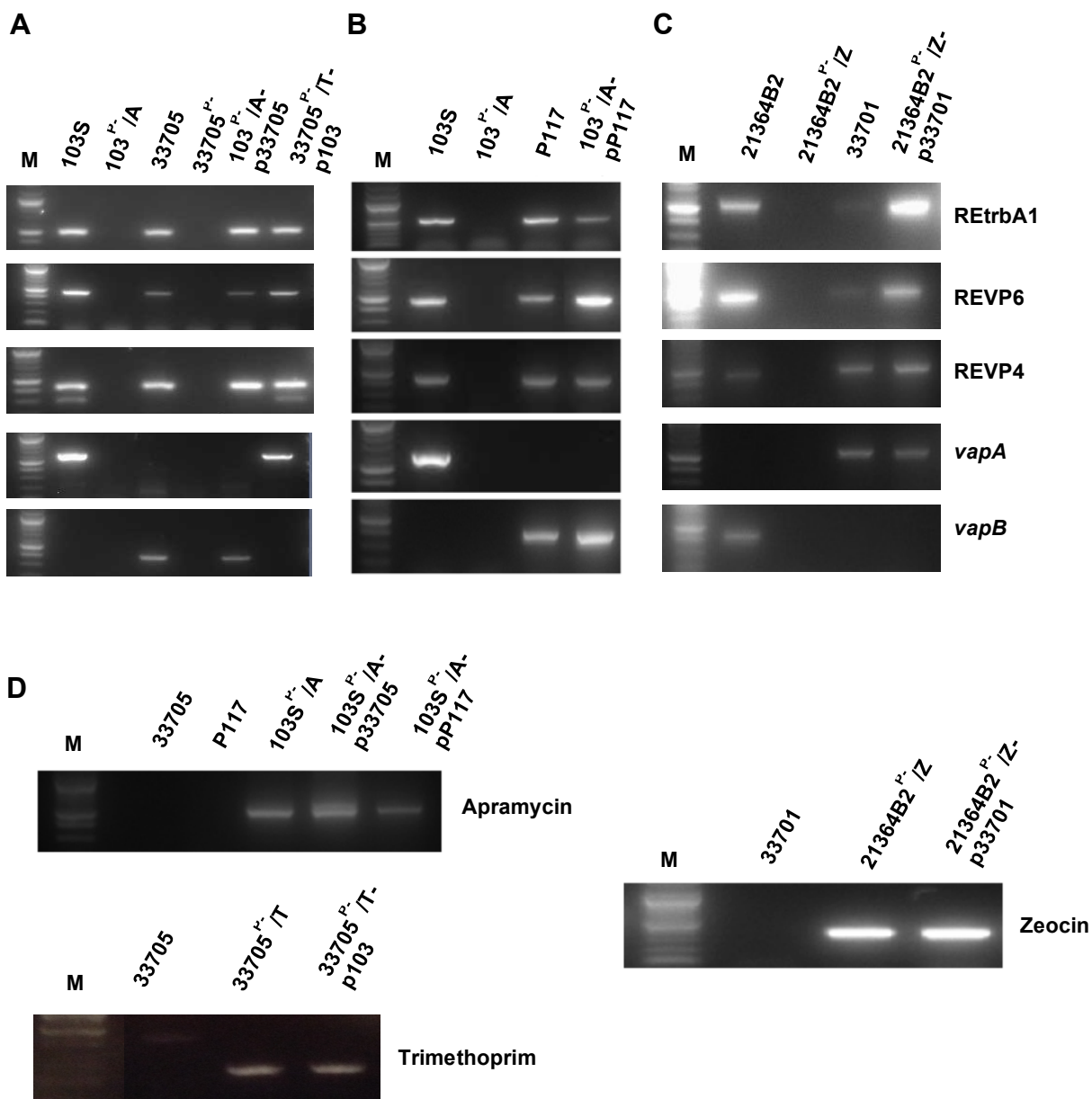

Supplement: Figure S1 [file sph005162162sf1.pdf]
